# Argon gas: a potential neuroprotectant and promising medical therapy

**DOI:** 10.1186/2045-9912-4-3

**Published:** 2014-02-17

**Authors:** Derek S Nowrangi, Jiping Tang, John H Zhang

**Affiliations:** 1 Department of Physiology, Loma Linda University School of Medicine, Risley Hall, Room 223, Loma Linda, CA 92354, USA; 2 Department of Neurosurgery, Loma Linda University School of Medicine, Loma Linda, CA, USA

**Keywords:** Argon, Xenon, Noble gas, Inert gas, Narcosis, Anesthesia, Neuroprotection, Organoprotection, Ischemia, Brain injury, Oxygen-Glucose deprivation, Traumatic brain injury, Middle cerebral artery occlusion, Tissue plasminogen activation, Argon plasma coagulation

## Abstract

Argon is a noble gas element that has demonstrated narcotic and protective abilities that may prove useful in the medical field. The earliest records of argon gas have exposed its ability to exhibit narcotic symptoms at hyperbaric pressures greater than 10 atmospheres with more recent evidence seeking to display argon as a potential neuroprotective agent. The high availability and low cost of argon provide a distinct advantage over using similarly acting treatments such as xenon gas. Argon gas treatments in models of brain injury such as in vitro Oxygen-Glucose-Deprivation (OGD) and Traumatic Brain Injury (TBI), as well as in vivo Middle Cerebral Artery Occlusion (MCAO) have largely demonstrated positive neuroprotective behavior. On the other hand, some warning has been made to potential negative effects of argon treatments in cases of ischemic brain injury, where increases of damage in the sub-cortical region of the brain have been uncovered. Further support for argon use in the medical field has been demonstrated in its use in combination with tPA, its ability as an organoprotectant, and its surgical applications. This review seeks to summarize the history and development of argon gas use in medical research as mainly a neuroprotective agent, to summarize the mechanisms associated with its biological effects, and to elucidate its future potential.

## Introduction

Argon gas is considered a small noble gas element that has been applied in a number of fields. It has been generally classified as a nonreactive or inert gas providing a view that it does not contain any biologically active characteristics. In fact, argon has demonstrated characteristics such as narcosis at hyperbaric pressures and more recently neuroprotective and organoprotective behaviors. Pharmaceutical drugs and surgical interventions have been widely accepted and commonly used methods of clinical treatments for a variety of applications such as the neuroprotection [1-4]. However, the high cost and development of new drugs and surgical techniques have exposed a need for new and easy to administer treatments. The application of argon as a medical gas presents a possible relief to this search and provides distinct advantages.

This review seeks to show the development of argon in the medical field with an emphasis on its ability as a neuroprotectant. In order to provide a foundation of argons role in the medical field, a summary of its discovery and characteristics will be first provided. We will then discuss the evidence suggesting the affirmative ability of argon as a neuroprotectant and highlight some evidence suggesting otherwise. In order to better understand how argon provides its biological actions, a review and discussion of the possible mechanisms of receptor interactions and apoptotic cellular pathways will be provided. Finally, we will consider the future of argon as a clinical therapy and other potential applications in the medical field.

### History and characteristics of argon

In 1785, Henry Cavendish isolated atmospheric gases in an attempt to characterize and quantify the substituents. In an attempt to combust the samples to find their reactivity profile, a small constituent of isolated gas samples believed to be nitrogen did not give the expected reaction and remained unchanged. After further analysis, it was concluded that the nonreactive nature of this gas sample was considered to be an error due to contamination [5]. The results were brought to attention again in 1894 when Lord Rayleigh and William Ramsay’s experimentation on atmospheric gases found that a similar amount of unreactive gas contained a unique set of characteristics. They confirmed that the unreactive gas was observed by Henry Cavendish and classified it to be a new element “Argon” [6].

Argon belongs to a family of elements located on the final column of the periodic table of elements termed “noble gases” which include the elements helium, neon, argon, krypton, xenon, and radon. The full electron valence shell of these elements prevents the formation covalent bonds and finding these gases in compound form remains a rare occurrence [7]. The inability to form strong covalent bonds with other elements develops an identity for the gases to be unreactive, grouping these gases under another term: inert gases. Though the term “inert gas” implies these gases have no active characteristics, several instances have been noted in which these gases are able to produce physical and biological effects [8]. The biological activity of argon can be attributed to its atomic structure interactions with enzymes and receptors. Though argon is unable to form strong covalent bonds to produce chemical reactions, it does have the capacity to interact with enzymes and receptors through charge-induced dipole and van der Waals interactions as a stabilizing component key in relating argon’s ability as an anesthetic and neuroprotectant [9,10].

Due to the similar structures and reactive natures of argon and xenon, a propensity to compare the two elements has appeared in a number of reports [11-13]. Xenon is one of the earliest and most widely investigated of the noble gases and is regarded as a potent anesthetic and a convincing neuroprotectant [14-17]. However, the limited availability (0.09 ppm) and high cost of xenon has prevented it from becoming a commonly used therapy option. The property of xenon as an inert gas has opened speculation of the other noble gases as possible alternatives with several of the noble gases revealing potentials in the medical field. In particular, argon displays attributes that position it as a likely alternative to xenon especially in the field of neuroprotection. Furthermore, the reasonably high availability (9340 ppm) of argon in the atmosphere allows for a low difficulty in obtaining the gas and makes argon relatively more cost effective as compared to xenon [18].

### Effects of argon as a medical gas

#### Argon as a narcotic agent

The first biological effect of argon gas can be found as a description of its narcotic capabilities represented in a study pertaining to the high-pressure effects of naturally occurring gases during deep sea diving. Previous observations noted that under high pressure with normal respiratory air, divers begin to develop narcotic symptoms of slowed mental cognition and psychological instability [19-21]. After isolating the gases, it was surmised that argon gas produces a strong narcotic effect at high pressures (>10 atm) as compared to helium and nitrogen, while xenon is able to produce narcotic symptoms at atmospheric pressure [22]. It was also theorized that the narcotic effects argon exerts is being created in a physical rather than a chemical manner due to its characteristic as inert gas lacking chemical reactions in the body [23].

The mechanism in which argon displays its anesthetic ability has been suggested to be from the stimulation of γ-aminobutyric acid type-A receptors (GABA_A_R) [24]. Though argons involvement has yet to be confirmed, other anesthetic gases such as nitrogen have also been suggested to stimulate the GABA_A_R as well [25]. Of further interest, other anesthetic gases such as xenon and nitrous oxide are described to antagonize N-methyl-D-aspartate receptors (NMDAR) to promote their narcotic effects [26,27]. The antagonism of NMDAR remains a plausible method for argon-induced anesthesia, but has yet to be established and is still under investigation. Additionally, dopamine release has been connected to both the activity of GABA_A_R and NMDAR, and a decrease in dopamine activity in the brain would promote a narcotic effect [28,29]. Argon gas treatments to rats found that levels of extracellular striatal dopamine are reduced at pressures exceeding 2.5 MPa (approximately 10 atm), suggesting a neurochemical method of which argon may present its anesthetic effects [30].

A number of gases including the noble gas family exhibit anesthetic tendencies, but greatly differ in the criteria required for their narcotic abilities to manifest [31]. Argon has been described as being an anesthetic agent, but is only able to exert these effects under hyperbaric pressures. Xenon has also been portrayed as a potent anesthetic, but manifests its narcotic effects at normobaric pressures. The noble gases helium and neon lack an observable anesthetic effect for reasons still under investigation [31,32]. The capacity of argon and xenon to produce anesthetic effects at different pressures may signify an important difference when considering their treatment potential in the medical field.

### Ischemic neuroprotective models

**Figure 1:**
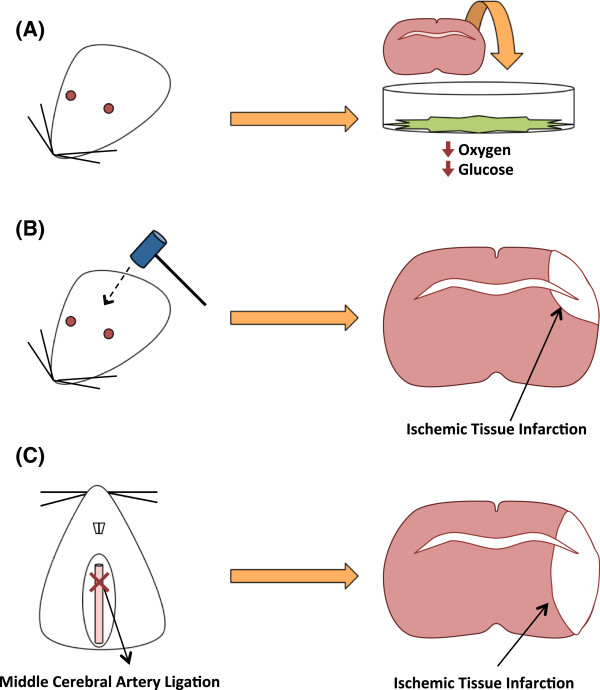
**Commonly used ischemic models of neuroprotection. (A)** An OGD model of neuroprotection places brain tissue into a medium that deprives it of oxygen and glucose in vitro. **(B)** The TBI model uses an apparatus to cause a forceful impact on the brain and results in ischemic tissue damage with treatments possible in vivo or in vitro. **(C)** The MCAO model ligates the middle cerebral artery to produce an ischemic infarction with treatments examined in vivo.

Of the potential uses argon in the medical field, studies pertaining to its ability as a neuroprotective agent have been most prominently examined. Investigations of neuroprotection seek to improve recovery of motor and behavioral functions of patients that have experienced neurological damage in cases such as but not limited to physical trauma [33,34]. Argon neuroprotective studies have largely been examined models of ischemic brain injuries in which the deprivation of essential nutrients such as glucose and oxygen to the brain may damage tissues and activate inflammatory and apoptotic pathways in surrounding tissues leading to neuronal death [35-39]. Oxygen-Glucose Deprived (OGD) environments, Traumatic Brain Injury (TBI), and the Middle Cerebral Artery Occlusion (MCAO) models are highly accepted methods of establishing ischemic brain injury treatments in rodent models and are the common methods of which argon neuroprotection treatments have been examined (Figure 1). An OGD model places a tissue of interest in a medium depriving the tissue of oxygen and glucose to simulate ischemic conditions in vitro [40]. The TBI model is a physical method of applied blunt force to the skull resulting in inflammatory and ischemic conditions with treatments possible in vitro and in vivo [41,42]. The MCAO model is considered an in vivo method of ligating the middle cerebral artery to simulate ischemic arterial blockage followed by reperfusion [43].

### Argon neuroprotection in vitro

A study by Jawad and colleagues aimed to compare the neuroprotective ability of the noble gases helium, neon, argon, and krypton to xenon in an Oxygen-Glucose Deprived (OGD) environment. Fetal mice cerebral cortices were exposed to an OGD medium and then treated to the noble gases (75% noble gas, 20% O_2,_ and 5% CO_2_). It was observed that argon and xenon improved neuronal survival while helium, neon, and krypton failed to significantly reduce neuronal death. Additionally, they observed that administering the gases without an OGD environment, is able to improve neuronal survival as compared to a control environment (75% N_2_ instead of noble gas) [18].

Loetscher and colleagues examined both models of OGD and TBI in vitro and found that all concentrations of the argon-oxygen gas mixtures tested (25-74% Ar, 21% O_2_, 5% CO_2_, N_2_ the rest) were effective in reducing cellular trauma injury to hippocampal brain slices of mice pups in both models. It was also noted that in the TBI model 50% argon exhibited the greatest reduction in injury but a concentration of 74% argon greatly attenuated this reduction in damage signifying a dose-dependent relationship of argon gas treatments. Additionally, it was demonstrated that delayed argon administrations given up to 3 hours after the insult were effective in reducing tissue damage [44]. Similarly, a study by Harris and colleagues found that argon (50% atm) and xenon (50% atm) gas are able to greatly attenuate the amount of injury up to 72 hours after TBI examined in vitro. The author’s note that though argon was able to reduce the total injury significantly, it was not as great of a reduction as found by Loetscher and colleagues [45].

### Argon neuroprotection in vivo

An in vivo examination by Ryang and colleagues using the MCAO model treated adult rats with 50% argon (50% O_2_) 1 hour following reperfusion of the middle cerebral artery. Reversals in tissue damage and a reduction in the edema-infarct volumes were observed up to 24 hours after reperfusion of the artery. The argon treated rats also demonstrated improved neurological function suggesting greater levels recovery and supporting the positive neuroprotective ability of argon. Furthermore, no significant differences of heart rate, blood pressure, and blood-gas measurements were monitored after argon inhalation signifying that normal physiological parameters were not disturbed [46].

Further supporting evidence for argon gas neuroprotection can be found in a study by Zhuang and colleagues aiming to compare noble gases treatments of helium, argon, and xenon to a control of nitrogen in a model of neonatal hypoxia-induced brain injury. Gas treatments (70% Ar/He/Xe and 30% O_2_) were given 2 hours after the hypoxic insult in which the right common carotid artery was ligated. They observed that cell morphology in the hippocampus right hemisphere was significantly restored for all treatment groups, but that argon was able to restore cell viability to the greatest extent. It was also noted that argon and xenon treatment groups displayed reductions of infarction sizes and that neurocognitive behavior results showed improvement for all gases [47].

Patients suffering a cardiac arrest may exhibit neurological damage by consequence of global brain ischemia and deregulation of elements such as coagulation and inflammatory factors [48]. Post-cardiac arrest, the sustained neurological damage may provide complications in the survival and recovery. In an effort to remedy this situation, Brücken and colleagues examined the effects argon gas provided after cardiac arrest in rats. The treatments were applied 1 hour after cardiac arrest resuscitation using 70% argon (30% O_2_). It was found that argon inhalation improved neurological scores with fewer damaged neurons observed in the cortex and hippocampal regions [49]. Similarly, Ristagno and colleagues found that treatments of 70% argon (30% O_2_) for 4 hours following porcine cardiac arrest resuscitation resulted in significantly improved neurological scores up to 72 hours after the injury [50].

### Negative neuroprotective effect

Though a number of ischemic brain injury studies have suggested a beneficial neuroprotective outcome due to argon gas exposure, a study by David and colleagues found that there remains a possibility for adverse consequences. A three-part examination found that positive neuroprotection was noted in cases of OGD and N-methyl-D-aspartate (NMDA) induced neuronal death, but negative effects were found in a model of MCAO. An in vivo treatment of 50% argon (50% O_2_) was applied 2 hours after a MCAO-induced injury in adult rats. Though the total brain damage was decreased, it was also found that argon increased the amount of damage found in the sub-cortex region with no improvements of behavior or motor functions. This is the first study to reveal a detrimental outcome of argon gas treatments in a model of neuroprotection [51].

The examination of the OGD-induced environment measured lactate dehydrogenase (LDH) as a marker of neuronal injury and found that argon-oxygen gas exposure (37.5% to 75% argon) decreased neuronal injury with the greatest reduction in damage found 3 hours after the insult. Additionally, NMDA injections were used to stimulate neuronal death with treatments of argon-oxygen gas (15-75% argon) given 1 hour after injection. Consistent with previous studies, treatments of 50% argon showed the maximal reduction of neuronal death in both the OGD and NMDA-induced cytotoxic environments. Though improvements were seen in the OGD environment and NMDA-induced cytotoxic models, the authors assess that argon gas would not be a suitable post-ischemic neuroprotective treatment for clinical use due to the negative outcome found in the MCAO examination, but also suggest that argon may be a useful neuroprotectant for other brain injuries such as TBI.

### Mechanisms

#### NMDAR mediated neuroprotection

As a neuroprotectant, little is understood about argons interactions to receptors and enzymes or the cellular pathways involved after its initial interactions, though there have been hints as to the involvement of NMDAR and GABA_A_R. Both NMDAR and GABA_A_R are widely accepted to be involved in a number of cell survival pathways and are also considered to be major targets of a number of anesthetics [52-55]. NMDAR are largely stimulated by glutamate and are considered to be excitatory neuronal receptors, while GABA_A_R are largely stimulated by γ-aminobutyric acid (GABA) and are considered to be inhibitory neuronal receptors [26,56]. It should also be considered that an increase of activity of one receptor type could result in lowering the activity of the other due to their similar but opposite involvement in their excitatory actions.

Previous reports have theorized that NMDAR interactions may be implicated in the mechanism of argon gas neuroprotection. An intrinsic pro-apoptotic mitochondrial pathway is activated in consequence of increased glutamate release and stimulation of NMDAR. The overstimulation of NMDAR produces a large influx of intracellular Ca^2+^, which is known to be a major cause of cytotoxic neuronal death [57-61]. The influx of Ca^2+^ triggers a rise in the pro-apoptotic Bax protein that competes with the anti-apoptotic signaling of Bcl-2 and Bcl-xL proteins [57,62,63]. Zhuang and colleagues found that the argon treated groups promoted an increased expression of the pro-survival protein Bcl-2 while having no effect on Bax or Bcl-xL expression in neuronal cells [47]. Furthermore, the attenuation of NMDA induced neuronal damage by argon gas observed by David and colleagues offers additional conjecture of NMDAR involvement [51].

Conversely, Harris and colleagues provide evidence that NMDAR are likely not involved in the neuroprotective activity of argon. Using glycine as a competitive inhibitor of NMDAR, no changes in argons neuroprotective behavior were discovered suggesting another mechanism in which argon activity is mediated. In addition, they also found through electrophysiological methods that the activity of TREK-1 potassium channels is not affected with argon application. Though the study didn’t expand on the cellular mechanisms of the neuroprotective behavior seen, it provides a clear representation of how NMDAR do not seem to be involved in argons neuroprotective behavior [45].

### GABA_a_R mediated neuroprotection

It can also be proposed that GABA_A_R may play a role in producing the neuroprotective effects of argon rather than NMDAR. Argon has been suggested to change GABA_A_R activity by binding to multiple discrete sites on the receptor [24]. However, this observation was suggested in relation to argons narcotic properties with no relationship to its neuroprotective properties being made. Several examinations of other drugs have observed that the stimulation of GABA_A_R has the ability produce neuroprotective results [64-66]. Of important consideration is that the involvement of GABA_A_R to argon signaling is in relation to its narcotic properties that are displayed only at hyperbaric pressures, while neuroprotection studies of argon are largely done at normobaric pressure.

### Mek-erk 1/2 pathway involvement

**Figure 2:**
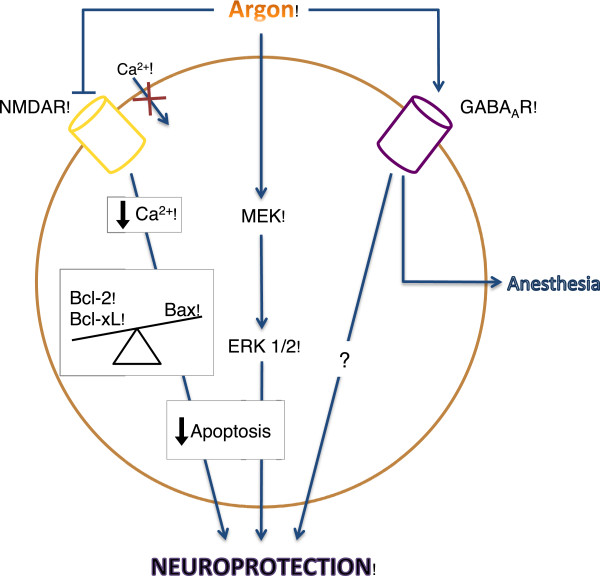
**Theorized argon neuroprotective pathways.** Suggested pathways still under investigation that may contribute to the neuroprotective effects of argon gas treatments in neuron cells include: NMDA receptor inhibition, direct stimulation of the MEK and ERK 1/2 anti-apoptotic pathway, and stimulation GABA_A_ receptor.

The neuroprotective behavior of argon may not be receptor mediated but may be the result of direct pathway participation. Fahlenkamp and colleagues noticed that argon treatments to cells in cultures increased expression of extracellular signal regulated kinases (ERK) 1/2 in neurons, microglia, and astrocyte cells. ERK 1/2 is a highly ubiquitous protein associated with a number of cellular pathways such as inflammatory and cell survival pathways depending on the methods of activation. It has been demonstrated that ERK 1/2 is largely regarded as a pro-death marker of neuronal cells, but through methods of transient stimulation can also be part of a pro-survival pathway [67]. Though these results did not investigate outcome results associated with the increase of ERK 1/2, it can be proposed that argon may be connected to the mitogen activated protein kinase (MAPK) kinase (MEK)-ERK 1/2 pathway in neuronal cells. Measurements found that argon does not change the level of phosphotyrosine phosphatases suggesting that an indirect cellular pathway is not stimulated. They suggest that because of the varied nature and independency from the cellular background of the MEK-ERK 1/2 pathway, argon may directly stimulate the pathway to assert its neuroprotective effects rather than through receptor mediation [68] (Figure 2).

### Future outlook

The use of argon gas for neuroprotective medical applications is a recent endeavor that has not expanded into clinical examinations as of yet. However, David and colleagues provided an examination on argons effects in combination with tissue plasminogen activator (tPA) that can be regarded as a indicator that an interest in its clinical application exists. In cases of acute ischemic stroke, the current and only approved method of clinical therapy has been the administration of tPA. At levels below 50% argon gas (25% oxygen and the rest nitrogen to complete mixture), argon decreased the catalytic and thrombolytic efficiency of tPA, but increased them at levels above 50% argon. These results seem to promote that argon gas may enhance the efficiency of tPA treatments and may foster support for use in clinical examination [69].

Besides argons use as a neuroprotectant, argon gas has also demonstrated organoprotectant traits. Irani and colleagues found that kidney recovery after storage with argon gas was improved as compared with mediums of xenon, nitrogen, or air. It was assessed that argon gas may provide a potential organoprotecive environment for organ transplants by preserving the quality and function of the kidney [70]. In addition, another study found that argon gas is able to protect the myocardium to infarction in cases of coronary artery occlusion and reperfusion. This study also examined the role of ERK 1/2 signaling, but did not find any changes in response to argon gas treatments [71]. It has also been found that argon and xenon (75% Ar/Xe, 20% O_2_, and 5% CO_2_) are able to limit apoptotic cell death when given up to 16 hours after inducing apoptosis to osteosarcoma cells in culture [72].

Another use of argon in the medical field has been as a surgical tool. Argon Plasma Coagulation (APC) is a non-contact technique that uses high frequency stimulation of argon plasma to cauterize surrounding tissues and prevent bleeding through coagulation around surgical sites. APC is an example of an early use of argon gas in a medical environment demonstrating an ability to effectively limit tissue damage as seen in surgical cases including skin, gastrointestinal, and neurosurgeries [73-77]. The positive feedback from the use APC has allowed for speculation on its aptitude as a suitable replacement for standardized coagulation techniques such as bipolar coagulation [78].

## Conclusion

The development of argon in medical research has originated from its ability as a narcotic agent to a gas with potential protective properties. The greater availability and low cost of argon provide a distinct advantage over xenon while the difference in the pressure required to exhibit narcotic properties allows for a variety of clinical applications not available to xenon. Ischemic brain injury models tend to show that argon is able to produce positive neuroprotective effects, though there also exists possible negative effects of argon gas therapies. Furthermore, the mechanisms in which argon exerts its neuroprotective behavior are poorly understood. Therefore, it can be evoked that an inadequate amount of data exists to correctly assess argons neuroprotective capability. Though studies investigating argon gas use in clinical therapies have yet to be examined, support for its use is evident through the combinational report with tPA. In addition, argon has garnered support as an organoprotectant and has shown progress as a surgical tool. Though much is still unknown about the effects and mechanisms of argon, a number of promising signs have been given to its future in the medical field with an emphasis found in its neuroprotective ability.

## Abbreviations

LDH: Lactate dehydrogenase; OGD: Oxygen glucose deprivation; TBI: Traumatic brain injury; MCAO: Middle cerebral artery occlusion; NMDA: N-Methyl-D-Aspartate; NMDAR: N-Methyl-D-Aspartate receptor; GABA: γ-Aminobutyric ccid; GABAAR: γ-Aminobutyric acid type-A receptor; tPA: Tissue plasminogen activator; APC: Argon plasma coagulation.

## Competing interests

The authors declare that they have no competing interests.

## Authors’ contributions

DSN - Role included reviewing manuscripts, review design, manuscript preparation, and manuscript editing. JT - Role included manuscript proof reading. JZ - Role included review design and manuscript proof reading. All authors read and approved the final manuscript.

## References

[B1] De Keyser J Sulter G Luiten PG Clinical trials with neuroprotective drugs in acute ischaemic stroke: are we doing the right thing? Trends Neurosci 1999 22 12 535 540 10.1016/S0166-2236(99)01463-0 10542428

[B2] Ito H Neuroprotective properties of propofol and midazolam, but not pentobarbital, on neuronal damage induced by forebrain ischemia, based on the GABAA receptors Acta Anaesthesiol Scand 1999 43 2 153 162 10.1034/j.1399-6576.1999.430206.x 10027021

[B3] Bilotta F Pharmacological perioperative brain neuroprotection: a qualitative review of randomized clinical trials Br J Anaesth 2013 110 Suppl 1 i113 i120 10.1093/bja/aet059 23562933

[B4] Tator CH Translational potential of preclinical trials of neuroprotection through pharmacotherapy for spinal cord injury J Neurosurg Spine 2012 17 1 Suppl 157 229 22985382 10.3171/2012.5.AOSPINE12116

[B5] Cavendish H Experiments on Air. By Henry Cavendish, Esq. F. R. S. & S. A Philos Trans R Soc Lond 1784 74 119 153 10.1098/rstl.1784.0014

[B6] Rayleigh L Ramsay W Argon, a new constituent of the atmosphere Proc R Soc Lond 1894 57 340–346 265 287

[B7] Christe KO Bartlett’s discovery of noble gas fluorides, a milestone in chemical history Chem Commun (Camb) 2013 49 41 4588 4590 10.1039/c3cc41387j 23575727

[B8] Ruzicka J Biological effects of noble gases Physiol Res 2007 56 Suppl 1 S39 S44 17552896 10.33549/physiolres.931300

[B9] Trudell JR Koblin DD Eger EI 2nd A molecular description of how noble gases and nitrogen bind to a model site of anesthetic action Anesth Analg 1998 87 2 411 418 9706942 10.1097/00000539-199808000-00034

[B10] Quillin ML Size versus polarizability in protein-ligand interactions: binding of noble gases within engineered cavities in phage T4 lysozyme J Mol Biol 2000 302 4 955 977 10.1006/jmbi.2000.4063 10993735

[B11] Gudmundsson JT Lieberman MA Ar + and Xe + velocities near the presheath-sheath boundary in an Ar/Xe discharge Phys Rev Lett 2011 107 4 045002 21867014 10.1103/PhysRevLett.107.045002

[B12] Schiwietz G Evidence for an ultrafast breakdown of the BeO band structure due to swift argon and xenon ions Phys Rev Lett 2010 105 18 187603 21231139 10.1103/PhysRevLett.105.187603

[B13] Kyrychenko A Waluk J Molecular dynamics simulations of matrix deposition. III. Site structure analysis for porphycene in argon and xenon J Chem Phys 2005 123 6 64706 10.1063/1.1997128 16122334

[B14] Ma D Neuroprotective and neurotoxic properties of the ‘inert’ gas, xenon Br J Anaesth 2002 89 5 739 746 10.1093/bja/89.5.739 12393773

[B15] Preckel B Molecular mechanisms transducing the anesthetic, analgesic, and organ-protective actions of xenon Anesthesiology 2006 105 1 187 197 10.1097/00000542-200607000-00029 16810011

[B16] Derwall M Xenon: recent developments and future perspectives Minerva Anestesiol 2009 75 1–2 37 45 18475253

[B17] Franks NP How does xenon produce anaesthesia? Nature 1998 396 6709 324 10.1038/24525 9845069

[B18] Jawad N Neuroprotection (and lack of neuroprotection) afforded by a series of noble gases in an in vitro model of neuronal injury Neurosci Lett 2009 460 3 232 236 10.1016/j.neulet.2009.05.069 19500647

[B19] Behnke AR Thompson RM Motley EP The psychologic effects from breathing air at 4 atmospheres pressure Am J Physiol Legacy Content 1935 112 3 554 558

[B20] Dudley SF Some atmospheric hazards encountered in naval life: (united services section) Proc R Soc Med 1935 28 9 1283 1292 19990380 10.1177/003591573502800946 PMC2205732

[B21] Haldane JBS Human life and death at high pressures Nature 1941 148 458 460 10.1038/148458a0

[B22] Lawrence JH Preliminary observations on the narcotic effect of xenon with a review of values for solubilities of gases in water and oils J Physiol 1946 105 3 197 204 PMC1393631 16991720

[B23] Behnke AR Yarbrough OD Respiratory resistance, oil–water solubility, and mental effects of argon, compared with helium and nitrogen Am J Physiol Legacy Content 1939 126 2 409 415

[B24] Abraini JH Gamma-aminobutyric acid neuropharmacological investigations on narcosis produced by nitrogen, argon, or nitrous oxide Anesth Analg 2003 96 3 746 749 table of contents 12598256 10.1213/01.ANE.0000050282.14291.38

[B25] Rostain JC Balon N Recent neurochemical basis of inert gas narcosis and pressure effects Undersea Hyperb Med 2006 33 3 197 204 16869533

[B26] Franks NP Lieb WR Molecular and cellular mechanisms of general anaesthesia Nature 1994 367 6464 607 614 10.1038/367607a0 7509043

[B27] Jevtovic-Todorovic V Nitrous oxide (laughing gas) is an NMDA antagonist, neuroprotectant and neurotoxin Nat Med 1998 4 4 460 463 10.1038/nm0498-460 9546794

[B28] Luo R Partridge JG Vicini S Distinct roles of synaptic and extrasynaptic GABAAreceptors in striatal inhibition dynamics Front Neural Circuits 2013 7 186 24324406 10.3389/fncir.2013.00186 PMC3840641

[B29] Ladepeche L Dupuis JP Groc L Surface trafficking of NMDA receptors: Gathering from a partner to another Semin Cell Dev Biol 2013 doi: 10.1016/j.semcdb.2013.10.005 10.1016/j.semcdb.2013.10.005 24177014

[B30] Balon N Opposing effects of narcotic gases and pressure on the striatal dopamine release in rats Brain Res 2002 947 2 218 24 10.1016/S0006-8993(02)02928-1 12176164

[B31] Koblin DD Minimum alveolar concentrations of noble gases, nitrogen, and sulfur hexafluoride in rats: helium and neon as nonimmobilizers (nonanesthetics) Anesth Analg 1998 87 2 419 24 9706943 10.1097/00000539-199808000-00035

[B32] Fowler B Ackles KN Porlier G Effects of inert gas narcosis on behavior–a critical review Undersea Biomed Res 1985 12 4 369 402 4082343

[B33] McConeghy KW A review of neuroprotection pharmacology and therapies in patients with acute traumatic brain injury CNS Drugs 2012 26 7 613 36 10.2165/11634020-000000000-00000 22668124

[B34] Liu R Neuroprotection targeting ischemic penumbra and beyond for the treatment of ischemic stroke Neurol Res 2012 34 4 331 7 10.1179/1743132812Y.0000000020 22643076

[B35] Russo R In search of new targets for retinal neuroprotection: is there a role for autophagy? Curr Opin Pharmacol 2013 13 1 72 7 10.1016/j.coph.2012.09.004 23036350

[B36] Zhang M Emerging roles of Nrf2 and phase II antioxidant enzymes in neuroprotection Prog Neurobiol 2013 100 30 47 23025925 10.1016/j.pneurobio.2012.09.003 PMC3623606

[B37] Neal JW Gasque P How does the brain limit the severity of inflammation and tissue injury during bacterial meningitis? J Neuropathol Exp Neurol 2013 72 5 370 85 10.1097/NEN.0b013e3182909f2f 23584204

[B38] Lee JM Zipfel GJ Choi DW The changing landscape of ischaemic brain injury mechanisms Nature 1999 399 6738 Suppl A7 14 10392575 10.1038/399a007

[B39] Durukan A Tatlisumak T Acute ischemic stroke: overview of major experimental rodent models, pathophysiology, and therapy of focal cerebral ischemia Pharmacol Biochem Behav 2007 87 1 179 97 10.1016/j.pbb.2007.04.015 17521716

[B40] Strasser U Fischer G Protection from neuronal damage induced by combined oxygen and glucose deprivation in organotypic hippocampal cultures by glutamate receptor antagonists Brain Res 1995 687 1–2 167 74 7583301 10.1016/0006-8993(95)00519-v

[B41] Prins M The pathophysiology of traumatic brain injury at a glance Dis Model Mech 2013 6 6 1307 1315 10.1242/dmm.011585 24046353 PMC3820255

[B42] Namjoshi DR Towards clinical management of traumatic brain injury: a review of models and mechanisms from a biomechanical perspective Dis Model Mech 2013 6 6 1325 1338 10.1242/dmm.011320 24046354 PMC3820257

[B43] Liu F McCullough LD Middle cerebral artery occlusion model in rodents: methods and potential pitfalls J Biomed Biotechnol 2011 2011 464701 21331357 10.1155/2011/464701 PMC3035178

[B44] Loetscher PD Argon: neuroprotection in in vitro models of cerebral ischemia and traumatic brain injury Crit Care 2009 13 6 R206 10.1186/cc8214 20017934 PMC2811924

[B45] Harris K Neuroprotection against Traumatic Brain Injury by Xenon, but Not Argon, Is Mediated by Inhibition at the N-Methyl-D-Aspartate Receptor Glycine Site Anesthesiology 2013 119 5 1137 1148 10.1097/ALN.0b013e3182a2a265 23867231

[B46] Ryang YM Neuroprotective effects of argon in an in vivo model of transient middle cerebral artery occlusion in rats Crit Care Med 2011 39 6 1448 53 10.1097/CCM.0b013e31821209be 21336110

[B47] Zhuang L The protective profile of argon, helium, and xenon in a model of neonatal asphyxia in rats Crit Care Med 2012 40 6 1724 30 10.1097/CCM.0b013e3182452164 22610177

[B48] Neumar RW Post-cardiac arrest syndrome: epidemiology, pathophysiology, treatment, and prognostication. A consensus statement from the International Liaison Committee on Resuscitation (American Heart Association, Australian and New Zealand Council on Resuscitation, European Resuscitation Council, Heart and Stroke Foundation of Canada, InterAmerican Heart Foundation, Resuscitation Council of Asia, and the Resuscitation Council of Southern Africa); the American Heart Association Emergency Cardiovascular Care Committee; the Council on Cardiovascular Surgery and Anesthesia; the Council on Cardiopulmonary, Perioperative, and Critical Care; the Council on Clinical Cardiology; and the Stroke Council Circulation 2008 118 23 2452 83 10.1161/CIRCULATIONAHA.108.190652 18948368

[B49] Brucken A Argon reduces neurohistopathological damage and preserves functional recovery after cardiac arrest in rats Br J Anaesth 2013 110 suppl 1 i106 i112 10.1093/bja/aes509 23393152

[B50] Ristagno G Post-resuscitation treatment with argon improves early neurological recovery in a porcine model of cardiac arrest Shock 2013 41 1 72 78 24088999 10.1097/SHK.0000000000000049

[B51] David HN Ex vivo and in vivo neuroprotection induced by argon when given after an excitotoxic or ischemic insult PLoS One 2012 7 2 e30934 10.1371/journal.pone.0030934 22383981 PMC3285153

[B52] Yang XR Involvement of MAPK pathways in NMDA-induced apoptosis of rat cortical neurons Sheng Li Xue Bao 2012 64 6 609 16 23258322

[B53] Jin J The blockade of NMDA receptor ion channels by ketamine is enhanced in developing rat cortical neurons Neurosci Lett 2013 539 11 5 23395831 10.1016/j.neulet.2013.01.034 PMC3602117

[B54] Ali Shah S Anthocyanins protect against ethanol-induced neuronal apoptosis via GABA receptors intracellular signaling in prenatal Rat hippocampal neurons Mol Neurobiol 2013 48 1 257 269 10.1007/s12035-013-8458-y 23645118

[B55] Yang L Sonner JM Anesthetic-like modulation of receptor function by surfactants: a test of the interfacial theory of anesthesia Anesth Analg 2008 107 3 868 74 10.1213/ane.0b013e31817ee500 18713898 PMC2668573

[B56] Mihic SJ Sites of alcohol and volatile anaesthetic action on GABA (A) and glycine receptors Nature 1997 389 6649 385 9 10.1038/38738 9311780

[B57] Randall RD Thayer SA Glutamate-induced calcium transient triggers delayed calcium overload and neurotoxicity in rat hippocampal neurons J Neurosci 1992 12 5 1882 95 1349638 10.1523/JNEUROSCI.12-05-01882.1992 PMC6575874

[B58] Choi DW Koh JY Peters S Pharmacology of glutamate neurotoxicity in cortical cell culture: attenuation by NMDA antagonists J Neurosci 1988 8 1 185 96 2892896 10.1523/JNEUROSCI.08-01-00185.1988 PMC6569373

[B59] Yeganeh F Neuroprotective effects of NMDA and group I metabotropic glutamate receptor antagonists against neurodegeneration induced by homocysteine in rat hippocampus: in vivo study J Mol Neurosci 2013 50 3 551 7 10.1007/s12031-013-9996-5 23564299

[B60] Lipton P Ischemic cell death in brain neurons Physiol Rev 1999 79 4 1431 568 10508238 10.1152/physrev.1999.79.4.1431

[B61] Dirnagl U Iadecola C Moskowitz MA Pathobiology of ischaemic stroke: an integrated view Trends Neurosci 1999 22 9 391 7 10.1016/S0166-2236(99)01401-0 10441299

[B62] Panaretakis T Activation of Bak, Bax, and BH3-only proteins in the apoptotic response to doxorubicin J Biol Chem 2002 277 46 44317 26 10.1074/jbc.M205273200 12193597

[B63] Li Y Han F Shi Y Increased neuronal apoptosis in medial prefrontal cortex is accompanied with changes of Bcl-2 and bax in a rat model of post-traumatic stress disorder J Mol Neurosci 2013 51 1 127 137 10.1007/s12031-013-9965-z 23381833

[B64] Costa C Coactivation of GABA (A) and GABA (B) receptor results in neuroprotection during in vitro ischemia Stroke 2004 35 2 596 600 10.1161/01.STR.0000113691.32026.06 14726544

[B65] Wei XW Neuroprotection of co-activation of GABA receptors by preventing caspase-3 denitrosylation in KA-induced seizures Brain Res Bull 2012 88 6 617 23 10.1016/j.brainresbull.2012.05.008 22613773

[B66] Dai J Activations of GABAergic signaling, HSP70 and MAPK cascades are involved in baicalin’s neuroprotection against gerbil global ischemia/reperfusion injury Brain Res Bull 2013 90 1 9 23041106 10.1016/j.brainresbull.2012.09.014

[B67] Subramaniam S Unsicker K ERK and cell death: ERK1/2 in neuronal death FEBS J 2010 277 1 22 29 10.1111/j.1742-4658.2009.07367.x 19843173

[B68] Fahlenkamp AV The noble gas argon modifies extracellular signal-regulated kinase 1/2 signaling in neurons and glial cells Eur J Pharmacol 2012 674 2–3 104 11 22094065 10.1016/j.ejphar.2011.10.045

[B69] David HN Modulation by the noble gas argon of the catalytic and thrombolytic efficiency of tissue plasminogen activator Naunyn Schmiedebergs Arch Pharmacol 2013 386 1 91 5 10.1007/s00210-012-0809-0 23142817

[B70] Irani Y Noble gas (argon and xenon)-saturated cold storage solutions reduce ischemia-reperfusion injury in a rat model of renal transplantation Nephron Extra 2011 1 1 272 82 10.1159/000335197 22470401 PMC3290848

[B71] Pagel PS Noble gases without anesthetic properties protect myocardium against infarction by activating prosurvival signaling kinases and inhibiting mitochondrial permeability transition in vivo Anesth Analg 2007 105 3 562 9 10.1213/01.ane.0000278083.31991.36 17717207

[B72] Spaggiari S Antiapoptotic activity of argon and xenon Cell Cycle 2013 12 16 2636 42 10.4161/cc.25650 23907115 PMC3865053

[B73] Miyazawa T Early experiences of haemostasis on brain tumour surgery with Argon Plasma Coagulation (APC) Acta Neurochir (Wien) 2000 142 11 1247 51 10.1007/s007010070021 11201639

[B74] Smythe A The effect of argon plasma coagulation ablation on esophageal motility and chemoreceptor sensitivity in Barrett’s esophagus patients Dis Esophagus 2010 23 6 445 50 10.1111/j.1442-2050.2010.01047.x 20236298

[B75] Goulet CJ In vivo evaluation of argon plasma coagulation in a porcine model Gastrointest Endosc 2007 65 3 457 62 10.1016/j.gie.2006.09.005 17321247

[B76] Min BH Feasibility and efficacy of argon plasma coagulation for early esophageal squamous cell neoplasia Endoscopy 2013 45 7 575 8 23801315 10.1055/s-0033-1344025

[B77] Ahn JY Clinical outcomes of argon plasma coagulation for the treatment of gastric neoplasm Surg Endosc 2013 27 9 3146 3152 10.1007/s00464-013-2868-9 23443483

[B78] Riegel T Comparative experimental study of argon plasma and bipolar coagulation techniques Acta Neurochir (Wien) 2006 148 7 757 62 discussion 762–3 10.1007/s00701-006-0770-0 16708172

